# The 3-min all-out test is valid for determining critical power but not anaerobic work capacity in tethered running

**DOI:** 10.1371/journal.pone.0192552

**Published:** 2018-02-14

**Authors:** Maria Carolina Traina Gama, Ivan Gustavo Masselli dos Reis, Filipe Antônio de Barros Sousa, Claudio Alexandre Gobatto

**Affiliations:** University of Campinas (UNICAMP), School of Applied Sciences, Limeira-SP, BRAZIL; Nanyang Technological University, SINGAPORE

## Abstract

The purpose of the study was to investigate if the 3-min all-out test (3MT) is valid for obtaining critical power intensity (CP) and the amount of work that can be performed above CP (W’) on non-motorized treadmills in tethered running. Eight physically active individuals (24 ± 3 years; 78.3 ± 8.7 kg; 179 ± 5 cm; 9.0 ± 2.5% body fat) performed four different efforts at constant intensity to exhaustion in order to determine CP and W’. The mechanical power values obtained were subsequently plotted with their corresponding time to exhaustion (limit time) for application of three mathematical models: power hyperbolic versus time limit (Hyp), linear power versus 1/time (P vs 1/t) and linear work versus time limit (Ԏ vs t). The 3MT test was carried out on the last day to determine end power (EP) and anaerobic capacity (WEP) using this methodology. EP value of 181.7 ± 52 was similar (p = 0.486) to 178.2 ± 61 (CP Hyp), 191.4 ± 55 (Ԏ vs t) and 188.3 ± 55 (P vs 1/t). WEP value of 17.9 ± 4.8 was not similar (p = 0.000) to 50.2 ± 15.3 (CP Hyp), 44.8 ± 8.7 (Ԏ vs t) and 45.5 ± 8.4 (P vs 1/t). Positive results (r = 0.78–0.98 and ICC = 0.88–0.99) of Pearson correlation and intraclass correlation (ICC–absolute agreement) were found for aerobic applications of conventional CP and 3MT. For anaerobic data, only the three models of conventional CP were correlated (r = 0.76–0.93); however, W’ from the three models was not correlated with WEP (r = 0.37–0.52). The results of this study suggest that 3MT in tethered running on non-motorized treadmills is a valid test for estimating CP aerobic parameters in a single day of application but not anaerobic parameters of W’.

## Introduction

The critical power test is a reliable tool to understand fatigue processes, evaluate human performance [1] and prescribe training as it is able to provide aerobic and anaerobic parameters using a non-invasive method [2]. The produced power is mathematically plotted against the respective exhaustion times to obtain the intensity of “fatigue threshold” [1], or critical power (CP), above which maximal oxygen uptake is evoked and the amount of work that can be performed above CP (W’) [2]. The evaluation needs different sessions of exhaustive effort (three or more intensities) [3]. This method was originally applied in three different models: hyperbolic–power versus time limit (Hyp), linear–power versus 1/time (P vs 1/t) and linear–work versus time limit (Ԏ vs t) [3]. Time limit represents the maximum time that the individual maintains a certain exercise intensity [1].

More recently, other researchers proposed a different methodology reasoning in critical power linear model theory (power versus 1/time), observing the mechanical power component over a maximal 180-s single effort [4]. The 3-minute all-out test (3MT) results can obtain the same parameters as the conventional CP test but using only one day of maximum exercise. In this model, the aerobic capacity is called EP (end power), and the W’ is called WEP. The original 3MT application [4] was performed on a cycle ergometer, and its reproducibility was confirmed by several authors on different ergometers [5, 6, 7]. The 3MT has already been applied as a critical velocity predictor in free running [2, 8], and the reliability of this test using measurements of tethered running power output on a non-motorized treadmill (NMT) has been recently demonstrated [7].

Mechanical power-based physical assessments in the specific movement of running are important in sports because they provide mechanical performance data that enables to determine the work (Ԏ) executed and allows physiological measures of energy expenditure during exercise [9, 10]. Furthermore, power output measure can be an essential tool to determine the individual potential of runners since it reflects a complete mechanical efficiency parameter–athletes who run faster using less force save energy outlay [7, 9]. In most sports that include running exercises, power output parameters are required to prescribe training, since their performance is a result of the muscle’s ability to respond as fast as it can, showing that running power plays a decisive role in several competitions, both in individual and team sports. Therefore, evaluations that determine aerobic and anaerobic parameters in running, measured by mechanical power output, are a reliable tool to identify powerful runners, the best training prescription, and performance prediction at different running intensities [7, 9].

Despite the power output’s importance for running performance [9], it is difficult to determine this variable maintaining the movement’s specificity when obtaining physiological and biomechanical information. Most protocols developed for obtaining physiological measures in runners are performed in the field or on motorized treadmill (MT), and aim to determine such intensities based on the velocity achieved by the individual [2, 8, 11]. Collecting information on the athlete’s power output and velocity capabilities provides a useful running efficiency index [7, 9, 12]. With this evaluative tool, which measures power output, in addition to physiological measures of aerobic and anaerobic capacity, it is possible to individually investigate the success or failure of trainings aimed at improving performance [9, 7]. The advantage of the 3MT test on NMT is that it is based on mechanical power output, which is the same power output parameter used in the traditional and original application of the CP concept [4]. In order to find an assessment method for runners that conserves movement specificity and is able to provide physiological parameters based on power output running in a single day, the objective of this study was to investigate if 3MT is valid for obtaining CP and W’ on NMT in tethered running. Similarly to the results found in cycle ergometer [4], our hypothesis is that EP and WEP from 3-min all-out test are valid for determining critical power and anaerobic work capacity, respectively, in tethered running.

## Materials and methods

### Subjects

Eight physically active men participated in this study (24 ± 3 years; 78.3 ± 8.7 kg; 179 ± 5 cm; 9.0 ± 2.5% body fat), performing the proposed tests after signing a written informed consent form approved by the Human Research Ethics Committee of the University of Campinas (protocol CAAE: 07716512.1.0000.5404), in accordance with the Declaration of Helsinki. The participants answered the International Physical Activity Questionnaire (IPAQ), in which the minimum score to classify them as “physically active” was used as inclusion criterion [13].

### Experimental procedures

The protocol consisted of seven days of visit to the laboratory. The subjects did not perform 3MT trials before the tests. They performed the tethered running familiarization two days prior to 3MT and CP sessions, consisting of ten sprints of ten seconds in the NMT with the aim of minimizing the learning effect during the acceleration phase. They then followed the protocol for determining anthropometric assessment, as previously described [12], and CP in four days. Subsequently, they carried out the 3MT test on the seventh day. Minimum 48-hour and maximum 72-hour intervals between tests were adopted [14]. Before all tests, the participants warmed up on a motorized treadmill running for five minutes at a 7.0 km/h speed. The warm-up, NMT ergometer familiarization, laboratory’s temperature and humidity conditions, and signal system to determine the mechanical parameters of force (N), velocity (m·s^−1^) and power output (W) followed the protocol established by previous studies in the same ergometer [7, 12] (for additional information see the Details of the methods in S1 File).

### Critical power test

**Fig 1:**
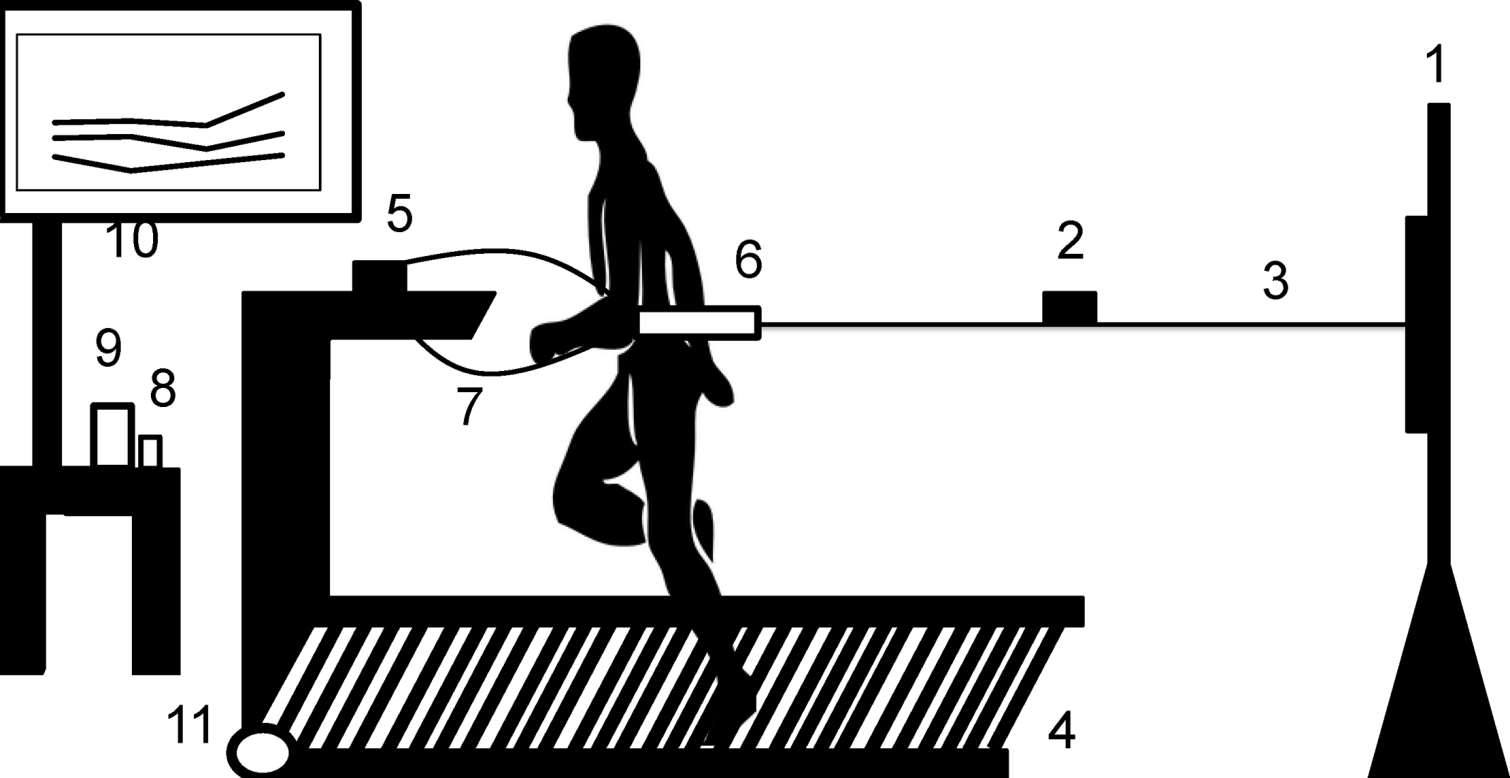
Schematic figure of the system developed on a non-motorized treadmill (NMT) for 3-min all-out (3MT) total power and critical power (CP) determination. 1. Height adjustment bar; 2. load cell; 3. steel cable with elastic cords (CP) or just steel cable (3MT); 4. NMT; 5. exhaustion apparatus; 6. belt attached to the steel cable and participant; 7. exhaustion apparatus security belt; 8. data acquisition module (DAC system); 9. signal amplifier; 10. computer; 11. hall effect sensor located on the treadmill’s front cylinder to determine the velocity that results from the connection with the DAC system.

In order to determine CP through the NMT-adapted protocol, the participants performed four efforts until exhaustion against different resistances in a randomized order. They ran tethered by a steel cable attached to an elastic cord (Fig 1). Resistance was increased between efforts by adding elastic cords (brand new elastics were used). The lowest resistance used three elastics, increasing one by one until the heaviest resistance (six elastics). The resistive force was measured by a signal acquisition system. Predictive efforts lasted between 2 and 15 minutes, and the participants maintained a constant intensity until exhaustion. An exhaustion apparatus was developed by the authors to keep runners at a targeted area, thus avoiding imposed load variation caused by change in position on the treadmill. When the runner was no longer able to sustain the established position on the treadmill, the exhaustion apparatus beeped, and the runner had five seconds to return to the established position. When the runner failed to return to his position due to exhaustion, the test was terminated [12].

### Determining the CP standard protocol’s aerobic and anaerobic parameters

In order to establish the intensity of each CP test predictive load, we calculated all graphs of horizontal power obtained in each of the four test sessions referred to as predictive load 1 (predictive 3 elastics), predictive load 2 (predictive 4 elastics), predictive load 3 (predictive 5 elastics) and predictive load 4 (predictive 6 elastics). The mechanical power values obtained were subsequently plotted with their corresponding time to exhaustion (limit time) for application of three mathematical models: power hyperbolic versus time limit (Hyp), linear power versus 1/time (P vs 1/t) and linear work versus time limit (Ԏ vs t). Through graphical analysis, it was possible to obtain the aerobic (CP) and anaerobic (W’) capacity parameters originating from the Hyp model (CP Hyp and W’ Hyp), the Ԏ vs t model (CP Ԏ vs T and W’ Ԏ vs t) and the P vs 1/t model (CP P vs 1/t and W’ P vs 1/t).

### 3MT test

In the 3MT test, the participants ran on a NMT (Fig 1) tethered to an adjustable-height pole by a steel cable attached to a load cell (CSL/ZL-250, MK Control and Instrumentation) [7]. The test session was only successful when the volunteer ran for the suggested 3 minutes non-stop. The participants were constantly encouraged, but they did not receive any information about the time during the test.

#### Determining 3MT aerobic and anaerobic parameters

The mechanical power output generated by each subject during the 3MT test was recorded and analyzed against time to find the aerobic capacity (EP) and anaerobic work capacity (WEP) values of the power output graph [7].

### Physiological analysis

Blood samples were collected from the ear lobe twice while at rest (LAC REP), and during 3MT and CP tests after 5 minutes post-exercise (LAC P5). The LAC P5 concentration was considered the peak value [12].

In the 3MT and predictive runs, the blood lactate concentrations were determined using the enzymatic method by spectrophotometry [7, 12]. Lactate concentration was determined at 340 nm against a calibration curve with 5, 10, 15 and 30 mM standards [15].

The participants’ heart rate (HR) was recorded (beat to beat) using Polar heart monitors (RS800CX). Data was recorded at rest (HR R) and immediately after the end of all tests (HR PT).

### Statistical analysis

The statistical analyses were performed using Statistica 7.0 (StatSoft, USA) software. Results are expressed as mean values, standard deviation (± SD) and coefficients of variation (CV%). CV% was obtained as SD normalized by the mean of a given variable. Initially, Shapiro-Wilk normality and Levene homogeneity tests were applied to identify the data’s characteristics. Based on the normality and homogeneity shown by data, the statistical methods suggested were parametric and adopted in all procedures.

Pearson correlations and intraclass correlation (ICC–absolute agreement) were subsequently applied. The parameters analyzed were the aerobic capacity values of CP Hyp, CP Ԏ vs 1/t and CP P vs 1/t and EP. The anaerobic data was W’ Hyp, W’ Ԏ vs t and W’ P vs 1/t from conventional CP, and WEP from 3MT. Repeated measures ANOVA and post hoc Newman-Keuls were used to compare the variables of aerobic and anaerobic capacity, mechanical data and acute physiological responses of heart rate (HR F) and peak blood lactate (LAC P) of all the assessment methods used. A significance level of p ≤ 0.05 was adopted in all hypothesis tests performed. The power was calculated using GPower software 3.1, and the effect size (ES) obtained in the statistical analysis was interpreted as suggested by Hopkins et al. [16]: ES < 0.2 considered trivial, 0.2–0.5 considered small, 0.6–1.1 considered moderate, 1.2–1.9 considered large, and > 2.0 considered very large. The Bland-Altman analysis (mean of differences ± 1.96*SD) was used to identify the existence or not of differences between aerobic and anaerobic parameters of critical power in 3MT [17]. The percentage error associated with the prediction of EP and WEP was calculated for the three CP and W’ models, respectively.

## Results

Mean values, standard deviation and CV% of aerobic and anaerobic data, effect size (ES), confidence interval (IC95%), as well as R^2^ of conventional critical power and 3MT applications, are shown in Tables 1 and 2. The 3MT test’s power output peak occurred in the first 7.3 ± 1.8 s, and this variable was stabilized at around 90 and 120 s of application.

**Table 1:** Aerobic capacity parameters derived from the application of the 3MT (EP) and conventional CP tests (critical power hyperbolic model–CP Hyp; critical power work model versus time–CP Ԏ vs t; critical power model versus 1/time–CP P vs 1/t), R^2^ of mathematical equations, coefficient of variation (CV), confidence interval (IC95%), the error % associated with the prediction of EP (Error %) and effect size (ES) (n = 8).

**AEROBIC CAPACITY–POWER OUTPUT (W)**				
	**EP**	**CP Hyp**	**CP Ԏ vs t**	**CP P vs 1/t**
**Mean**	181.7	178.2	191.4	188.3
**SD**	52	61	55	55
**IC 95%**	43.4	50.9	46	46
**R**^**2**^	0.78	0.98	0.95	0.95
**CV%**	30.4	35.8	31.3	32.5
**Error %**	-	24.1	24.2	21.3
**EFFECT SIZE**				
	**EP**	**CP Hyp**	**CP Ԏ vs t**	**CP P vs 1/t**
**EP**	-	0.06	0.18	0.12
**CP Hyp**	0.06	-	0.22	0.17
**CP Ԏ vs t**	0.88	0.22	-	0.23
**CP P vs 1/t**	0.12	0.17	0.23	-

*The significance criterion adopted was p ≤ 0.05.

**Table 2:** Anaerobic capacity parameters derived from the application of the 3MT (WEP) and conventional CP tests (W’ Hyp; anaerobic work capacity work model versus time–W’ Ԏ vs t and anaerobic work capacity model versus 1/time–W’ P vs 1/t), R^2^ of mathematical equations, coefficient of variation (CV), confidence interval (IC95%), the error % associated with the prediction of WEP (%Er-WEP) and effect size (ES) (n = 8).

**ANAEROBIC WORK CAPACITY (kJ)**				
	**WEP**	**W’ Hyp**	**W’ Ԏ vs t**	**W’ P vs 1/t**
**Mean**	17.9	50.2*	44.8*	45.5*
**SD**	4.8	15.3	8.7	8.4
**IC 95%**	4	12.8	7.2	7
**R**^**2**^	0.78	0.98	0.95	0.95
**CV%**	27.8	33.3	19.9	18.8
**Error %**		245.1	204.5	182.5
**EFFECT SIZE**				
	**WEP**	**W’ Hyp**	**W’ Ԏ vs t**	**W’ P vs 1/t**
**WEP**	-	3.07	3.92	4.18
**W’ Hyp**	3.07	-	0.45	0.43
**W’ Ԏ vs t**	3.92	0.45	-	0.08
**W’ P vs 1/t**	4.18	0.43	0.08	-

* Significant difference in relation to WEP’

For aerobic capacity data, repeated measures ANOVA did not reveal statistical differences among CP Hyp, CP Ԏ vs t, CP P vs 1/t and EP. However, in the anaerobic capacity data, the statistical test indicated significant differences (Table 2), with the same results to W’ Hyp, W’ Ԏ vs t and W’ P vs 1/t, but higher if compared to the 3MT test’s WEP.

In response to the increase in resistance, the hyperbolic relationship between strength or power output against time in conventional critical power test was observed, but this condition did not occur in the velocity variable (Table 3) (for additional information see the Raw data in S2 File).

**Table 3:** Mean and standard deviation of the power output, force, velocity and time limit variables of each predictive load from the conventional CP test (n = 8).

	PREDICTIVE 6 ELASTICS	PREDICTIVE 5 ELASTICS	PREDICTIVE 4 ELASTICS	PREDICTIVE 3 ELASTICS
**Power Output(W)**	527.4±117*	418.7±97*	293.5±42*	253.1±43*
**Time Limit (s)**	164±68*	242.5±102*	453.5±137*	628.6±138*
**Velocity (m/s)**	2.57±0.3	2.46±0.3	2.06±0.2^●^	2.0±0.2^●^
**Force (N)**	191.3±27*	162.3±27*	136.5±18*	119.1±11*

*Statistic difference from the other intensities (p ≤ 0.05)

^●^Statistic difference from 3 and 4 predictive elastics (p ≤ 0.05)

The physiological parameters (blood lactate and HR) of the applications of the 3MT test and CP predictive loads did not show significant differences (Table 4). The sample power (1-β err prob) were 0.98 and 0.99 for aerobic and anaerobic parameters, respectively.

**Table 4:** Mean ± standard deviation of physiological parameters of lactate peak (LAC P) and heart rate (HR PT) derived from the application of 3MT and predictive load tests to obtain conventional CP (predictive 3 elastics, predictive 4 elastics, predictive 5 elastics, predictive 6 elastics) (n = 8).

PHYSIOLOGICAL PARAMETERS						
		3MT	PREDICTIV 3 ELASTICS	PREDICTIV 4 ELASTICS	PREDICTIVE 5 ELASTICS	PREDICTIVE 6 ELASTICS
**LAC P (mmol·L**^**−1**^**)**	**Mean**	11.2	10.5	10.6	13.2	11.7
	**SD**	3.3	4.1	3.4	3	3.2
**HR PT (bpm)**	**Mean**	177	180	179	179	179
	**SD**	7	11	10	9	8

*The significance criterion adopted was p ≤ 0.05.

**Fig 2:**
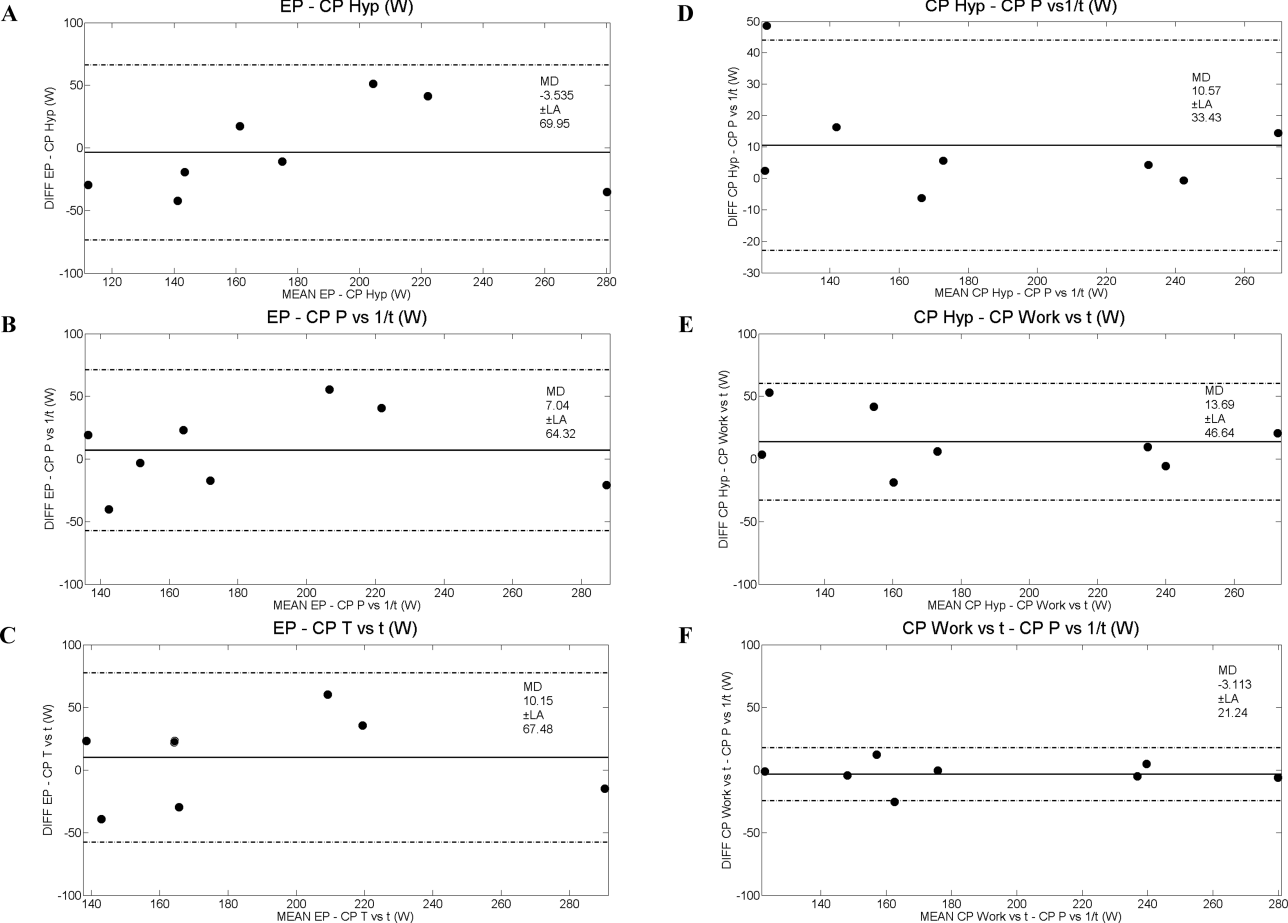
Limits of agreement among aerobic parameters of CP Hyp, CP Ԏ vs t, CP P vs 1/t and EP through the Bland and Altman analysis [17]. W, Watts. Diff, difference among values of aerobic parameters. A (EP—CP Hyp), B (EP- CP P vs 1/t), C (EP—CP Ԏ vs t), D (CP Hyp—CP P vs 1/t), E (CP Hyp—CP Work vs t) and F (CP Work vs t—CP P vs 1/t).

**Fig 3:**
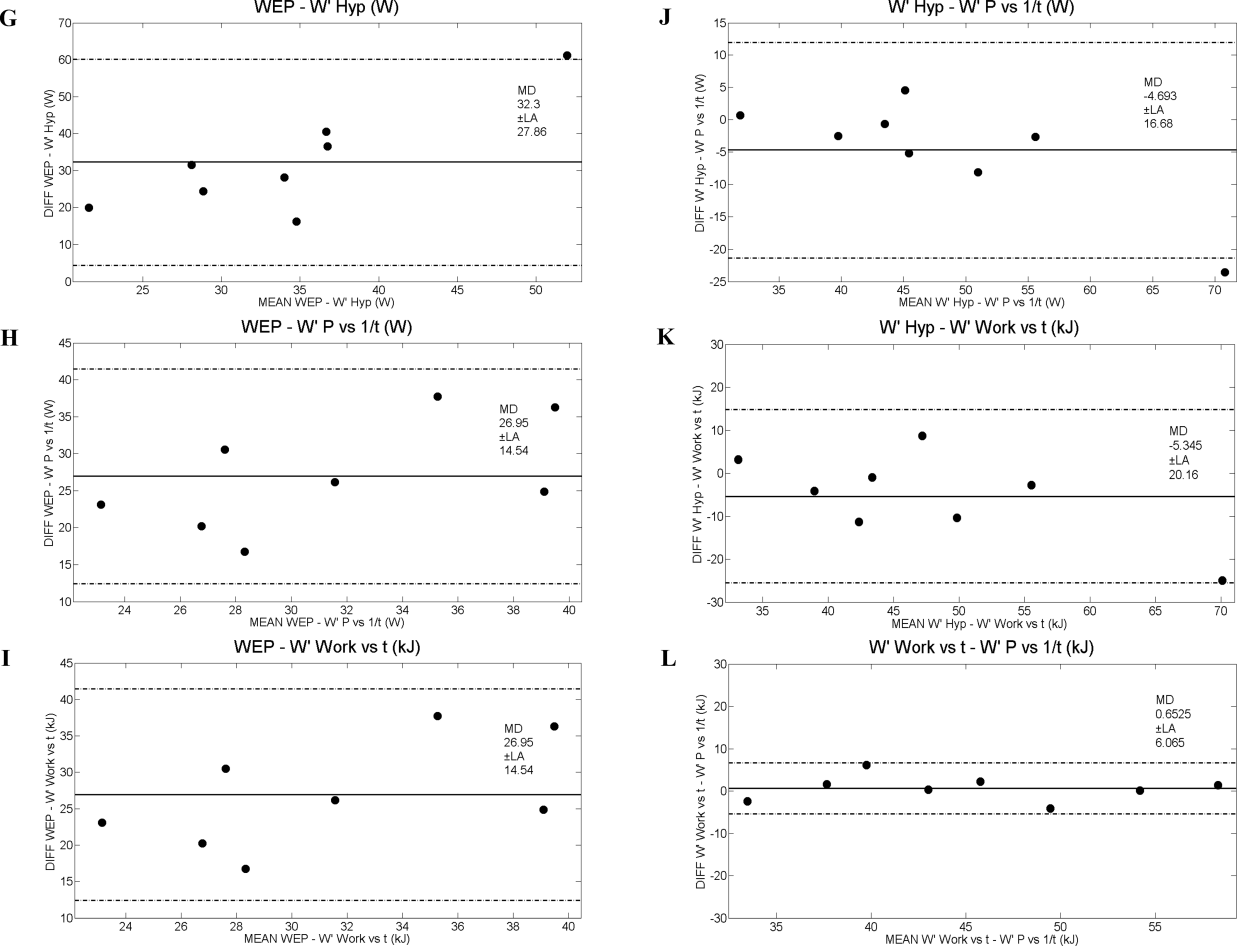
Limits of agreement among aerobic parameters of W’ Hyp, W’ Ԏ vs t and W’ P vs 1/t, compared to WEP through the Bland and Altman analysis [17]. kJ, Kilojoule. Diff, difference among values of anaerobic parameters. G (WEP—W’ Hyp), H (WEP–W’ P vs 1/t), I (WEP–W’ Work vs t), J (W’ Hyp–W’ P vs 1/t), K (W’ Hyp–W’ Work vs t) and L (W’ work vs t–W’ P vs 1/t).

**Fig 4:**
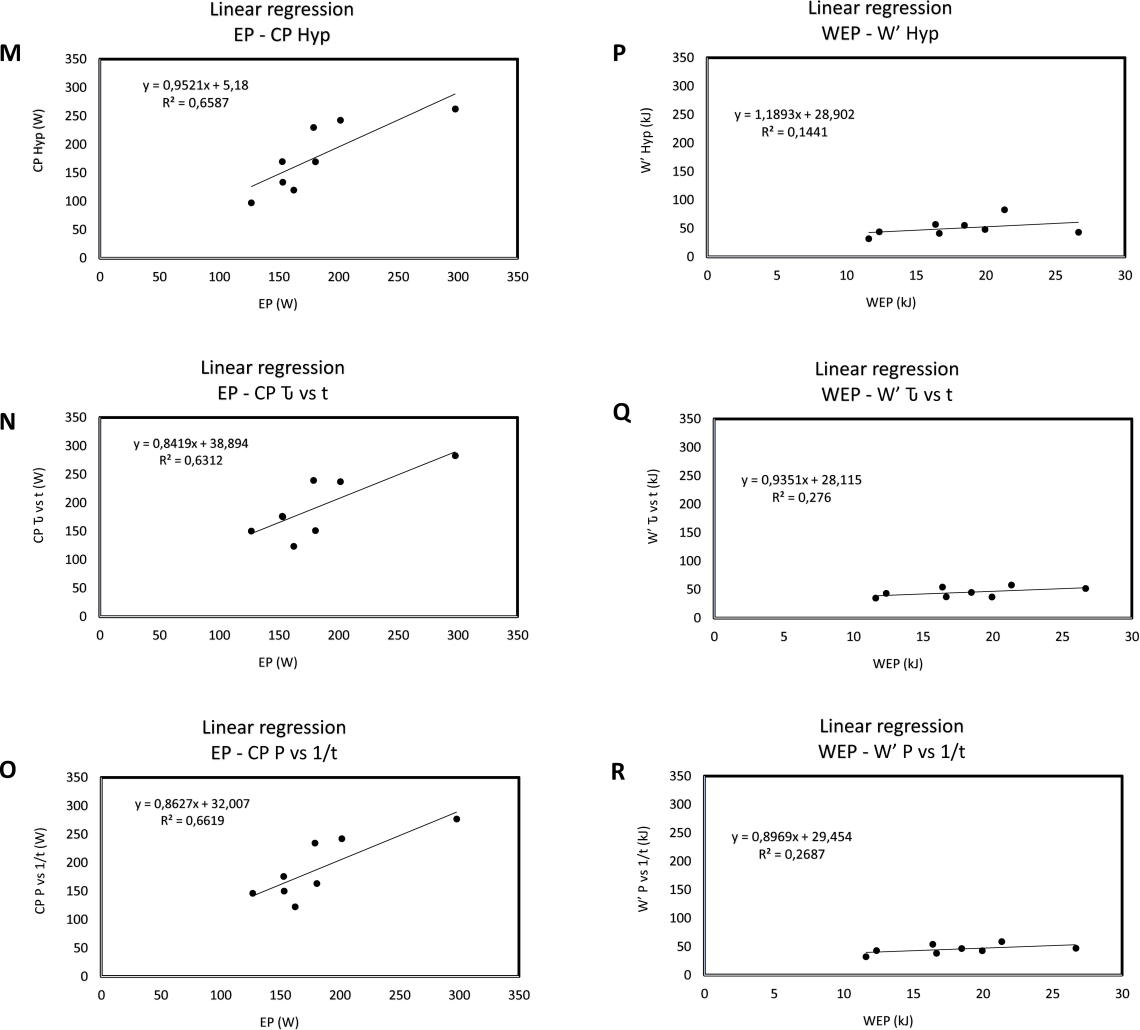
Linear regression among aerobic parameters of CP Hyp, CP Ԏ vs t, CP P vs 1/t and EP, and anaerobic parameters of W’ Hyp, W’ Ԏ vs t and W’ P vs 1/t, compared to WEP. M (EP—CP Hyp), N (EP—CP Ԏ vs t), O (EP- CP P vs 1/t), P (WEP—W’ Hyp), Q (WEP–W’ Ԏ vs t) and R (WEP–W’ P vs 1/t).

Table 5 shows positive correlations (Pearson and absolute agreement ICC) for aerobic parameters, whereas positive correlation for anaerobic capacity was found only for those parameters obtained by the conventional CP test. The Bland Altman analyses of aerobic and anaerobic data from conventional critical power and 3MT applications are shown in Figs 2 and 3, respectively. Linear regression among aerobic parameters of CP Hyp, CP Ԏ vs t, CP P vs 1/t and EP, and anaerobic parameters of W’ Hyp, W’ Ԏ vs t and W’ P vs 1/t, compared to WEP are shown in Fig 4.

**Table 5:** Results of Pearson correlation and absolute agreement ICC between aerobic and anaerobic parameters from the application of the 3MT (EP–WEP) and conventional CP tests (CP Hyp, CP Ԏ vs t and CP P vs 1/t—W’ Hyp, W’ Ԏ vs t and CP P vs 1/t) (n = 8).

		**AEROBIC PARAMETERS (W)**							
		**EP**		**CP Hyp**		**CP Ԏ vs T**		**CP P vs 1/t**	
		**Pearson**	**ICC**	**Pearson**	**ICC**	**Pearson**	**ICC**	**Pearson**	**ICC**
**EP**	**R**	–	–	0.81*	0.96*	0.79*	0.90*	0.81*	0.88*
**CP Hyp**	**R**	0.81*	0.96*	–	–	0.92*	0.99*	0.96*	0.90*
**CP Ԏ vs t**	**R**	0.79*	0.90*	0.92*	0.99*	–	–	0.98*	0.97*
**CP P vs 1/t**	**R**	0.81*	0.88*	0.96*	0.90*	0.98*	0.97*	–	–
		**ANAEROBIC PARAMETERS (kJ)**							
		**WEP**		**W’ Hyp**		**W’ Ԏ vs T**		**W’ P vs 1/t**	
		**Pearson**	**ICC**	**Pearson**	**ICC**	**Pearson**	**ICC**	**Pearson**	**ICC**
**WEP**	**R**	–	–	0.37	0.00	0.52	0.00	0.51	0.00
**W’ Hyp**	**R**	0.37	0.00	–	–	0.76*	0.00	0.90*	0.68
**W’ Ԏ vs t**	**R**	0.52	0.00	0.76*	0.00	–	–	0.93*	0.10
**W’ P vs 1/t**	**R**	0.51	0.00	0.90*	0.68	0.93*	0.10	–	–

*The significance criterion adopted for the correlations was p ≤ 0.05.

## Discussion

The objective of this study was to investigate if 3MT is valid for obtaining CP and WEP on NMT in tethered running. The main finding of this research suggests that 3MT is a valid test for estimating CP aerobic parameters but not W’. Besides being performed in a single session, this research protocol maintains movement specificity while providing the instruments to acquire dynamic strength, speed and power data in runners, which are essential for running.

The feasibility of CP application was confirmed by the R^2^ values presented (Table 1) [18, 19] and by the stipulated duration restriction of 2 to 15 minutes (Table 3), as proposed by other authors [20, 21]. Regarding 3MT application, its reproducibility and validity were verified in other ergometers [4, 22, 23] and shown to be reliable for mechanical power output parameters in NMT [7]. The mean time for achieving a power output peak and stabilization values (90–120 s) of this research also corroborated with other studies that used 3MT in different ergometers [22, 23, 24, 25, 26, 27].

When comparing both applications, the responses of physiological markers of LAC P and HR PT (Table 4) showed no significant difference between methodologies. The post-test lactate concentration indicates participation of anaerobic lactic metabolism [3, 28], which strengthens anaerobic measures, despite not assuring maximal effort.

The fact that the aerobic capacities of the 3MT and conventional CP on NMT were not statistically different (Table 1) and showed a high-correlation value and mutual reliability ICC (Table 5) ensures consistency of both applications and strengthens the ergometer model. Furthermore, the Bland Altman analyses corroborated with this assumption, with mean difference (MD) values close to zero (Fig 2). Additionally the fit presented in the linear regressions of PE in relation to the three CP models were acceptable (Fig 4). In this context, 3MT has the advantage of requiring only one test session in comparison to conventional CP. Additionally, in mathematical terms, this application minimizes conventional CP errors due to linear or non-linear mathematical treatment, since the variables are directly obtained only by recording the produced mechanical power [4]. Although the mean Error—EP is moderate (~23.2%), this should not be a problem since they are different methodologies, since EP its directly derived from the mechanical power of the final 30s of the test and the values from the conventional CP are mathematical models. The other statistical results support the use of EP to estimate CP because they present consistency, reliability and correlation among each other. The Error—WEP (~210.7%) value does not support the estimation of W’ by such methodology, corroborating with the other statistical results (Figs 3G, 3H, 3I, 4P, 4Q and 4R). Since it is important to ensure spontaneity and specificity during this kind of evaluation [29, 30] and both applications on NMT tethered running are able to provide it, the use of 3MT seems to be more applicable due to the advantages mentioned above.

Until now, no study has analyzed physiological data derived from mechanical power output units through the application of CP or 3MT tests in tethered running. Furthermore, the anaerobic data used for this modality is typically quantified in units of anaerobic distance of the critical velocity test application [2, 8,11]. However, recent studies have shown a positive correlation between runner power output and anaerobic capacity measured in NMT using an alternative Maximal accumulated oxygen deficit (MAOD) method, which is a test that has a consolidated physiological robustness [29]. Therefore, the power output measure seems to be appropriate for the anaerobic parameter because it considers the force application. Despite these benefits, WEP and W’ showed poor agreement in the current investigation.

The CP concept refers to the tolerable duration of severe-intensity exercise and W’ refers to the curvature constant of hyperbolic model [1]. Theoretically, W’ and WEP reflect the amount of work that the individual is able to perform with ATP-CP and glycogen finite energy reserves [31, 32], and recent studies suggest that changes in CP influence W’, since both may be affected by hypoxia and hyperoxia conditions [1, 33, 34]. Furthermore, load profile may affect WEP magnitude [33].

Previous studies discussed that high-level and homogenous individuals adopt a strategy that saves energy to use at the end of the test when performing submaximal tests [23, 35, 36], this is not the case in our sample, which consisted of active males with CV% in each test of around 30%. These characteristics may prevent the adoption of a strategy in this study, not allowing the EP to be overestimated and the WEP to be underestimated. Furthermore, an inspection of the power output’s kinetics and other physiological variables, such as blood lactate concentration, was conducted as recommended in order to reduce this problem when using 3MT.

A factor that could influence the anaerobic component of critical power models is ecological validity. Galbraith et al. [8] and Triska et al. [37] discussed that differences between anaerobic parameters of critical power obtained in laboratory and in field are caused by maintenance of a fixed speed or cadence in the laboratory in contrast to spontaneous variation in the field. Furthermore, our study was performed in a NMT where runners could vary force and velocity at their own will. Possibly, 180 s of test is not sufficient to deplete anaerobic metabolism reserves [4] in 3MT, and tests involving maximum sprints usually disregard the initial acceleration phenomenon. A study using measurements of oxygen consumption and critical velocity applied on a treadmill reported that faster outputs accelerate the use of oxidative metabolism and seem to spare the runner’s anaerobic energy [38].

In the original study of 3MT application versus linear models of CP in cycle ergometer, WEP was not statistically different from W’ [4], but in six of ten participants, capacity data of the conventional model was considerably higher than that of the 3MT. The reference authors suggest that this actually happened due to methodological characteristics resulting from uncontrolled acceleration of 3MT on the cycle ergometer. They also indicate that there was complete exhaustion of anaerobic metabolism in 3MT, since the stabilization of oxygen consumption was similar in EP and on the application of a ramp test. Another study demonstrated that the W' value does not change with prior exercise and effort at intensities above or below EP [26]. However, no similar results were found when using different cadences in isokinetic cycle ergometer, presenting dependence protocol relation [33]. Additionally, Bertram et al. [35] also found disagreement between both protocols’ parameters while using six high-level cyclists that performed six maximum bouts in two days. Factors such as proper cadence and rest intervals between bouts may be important to the quality of 3MT parameters, but this is yet to be studied. Validity of anaerobic parameters of both tests seems debatable since the anaerobic reserves theory does not seem to be an independent fixed amount. Factors such as the initial acceleration, activity and amount of muscle fibers recruited seem to affect this amount of energy [4, 23, 33, 39].

A possible study limitation is the light frontal tilt of the runner’s body as a result of treadmill friction, causing a difference in position of the body’s center of mass. Additionally, the NMT used here does not account for the vertical force component. On the other hand, the ergometer allows spontaneous development of velocity and power output, which is not possible on a motorized treadmill.

It is important to clarify that the 3MT test was performed after accomplanished the conventional CP test. Considering that the prediction times for the protocol varied between 164–628 seconds, certainly the participants were quite familiar with non-motorized treadmill running exercise. Also, Vanhatalo et al. [7] suggests that the initial phase of acceleration during 3MT may interfere with WEP values. The choice of 10s sprints was aimed at avoiding the learning effect during the acceleration phase and adapting the subjects to the 3MT protocol. In fact, in the original article Vanhatalo et al. [7] performed the adaptation to the 3MT test, however, by the considerations above, it must not to be consided the lack of specific familiarization to the whole 3-minute test a limitation to the present study. Additionally, in a previous study of our group, the reliability of the 3MT test in NMT tethered running was demonstrated [7].

## Conclusion

In conclusion, the main finding of this research suggests that 3MT is a valid test estimating aerobic parameters of CP in a single application day but did not the anaerobic parameter of W’. Furthermore, we showed that the conventional application of CP and 3MT, when performed in tethered running on NMT, can be a viable methodology for obtaining mechanical power units in a specific ergometer for runners. The 3MT test has unquestionable advantages such as conclusion in a single 3-minute session and being able to provide aerobic and anaerobic physiologic parameters.

## Supporting information

S1 File This is the S1 Supplementary file. The supplementary file provides details of the methods.
Details of the methods in S1 File. The details of the methods provide additional information about the ergometer and the mechanical and blood tests.
References in S1 File. The references present the essential manuscripts of methodological details.
(DOCX)

S2 File This is the S2 Raw data. The raw data presents the raw results from 3MT and conventional critical power (n = 8).
Table A in S2 file. Mean ± standard deviation of physiological parameters of heart rate (HR PT) derived from the application of 3MT and predictive load tests to obtain conventional CP (predictive 3 elastics, predictive 4 elastics, predictive 5 elastics, predictive 6 elastics) (n = 8).
Table B in S2 File. Mean ± standard deviation of physiological parameters of lactate peak (LAC P) derived from the application of 3MT and predictive load tests to obtain conventional CP (predictive 3 elastics, predictive 4 elastics, predictive 5 elastics, predictive 6 elastics) (n = 8).
Table C in S2 File. Mean and standard deviation of the velocity of each predictive load from the conventional CP test (n = 8).
Table D in S2 File. Aerobic capacity parameters derived from the application of the 3MT (EP) and conventional CP tests (critical power hyperbolic model–CP Hyp; critical power work model versus time–CP Ԏ vs t; critical power model versus 1/time–CP P vs 1/t) (n = 8).
Table E in S2 File. Mean and standard deviation of the power output of each predictive load from the conventional CP test (n = 8).
Table F in S2 File. Mean and standard deviation of the force of each predictive load from the conventional CP test (n = 8).
Table G in S2 File. Mean and standard deviation of the time limit of each predictive load from the conventional CP test (n = 8).
Table H in S2 File. Anaerobic capacity parameters derived from the application of the 3MT (WEP) and conventional CP tests (W’ Hyp; anaerobic work capacity work model versus time–W’ Ԏ vs t and anaerobic work capacity model versus 1/time–W’ P vs 1/t) (n = 8).
(XLSX)
