# Supplementary material for: The 3-min all-out test is valid for determining critical power but not anaerobic work capacity in tethered running
Source: PLoS One. 2018 Feb 14;13(2):e0192552. doi: 10.1371/journal.pone.0192552 (PMC5812641; doi:10.1371/journal.pone.0192552)
Supplement: S1 File — The supplementary file provides details of the methods. Details of the methods in S1 File. The details of the methods provide additional information about the ergometer and the mechanical and blood tests. References in S1 File. The references present the essential manuscripts of methodological details. (DOCX) [file pone.0192552.s001.docx]

**S1 Supplementary file**

**S1 Details of the methods**

The velocity of individuals in the two tests on NMT has been determined by a Hall Effect sensor and a magnet, located in the anterior cylinder (roller) of this ergometer. The data acquisition frequency of strength in all tests and velocity of the NMT protocols was at 1000 Hz. Importantly, the equipment calibration for force measurements was performed before each test with barbells of known weight and subsequently processed in newton. The horizontal vector of force data has been obtained in the form of a matrix of electric potentials difference (mV), allowing conversion into force (N) units by means of a linear calibration equation. This calibration has been performed by capturing signals from known weights in units of force (kgf). Each weight used had its respective load cell for signal captured for five seconds. The average value of this signal provides the information that composed the linear regression. By using the formula generated for this line, it was possible to transform the load cell signal at every millisecond for the actual tests [1].

Both load cell as the Hall Effect sensor output signals were modulated by a signal conditioner (USB-6008, National Instruments®). These have been captured by a computer with specific software (Lab View Signal Express 2009, National Instruments®) in the frequency cited. Data obtained through this system, during the tests, have been subsequently transferred to MatLab (MatLab® R2008a, MathWorkstm), where the information in millivolts (mV), generated by both sensors was treated, to be then interpreted and transformed into units of force (Newton) and velocity (m/s) [[1](#_ENREF_1), [2](#_ENREF_2)].

For data treatment of velocity determining on NMT (m/s) the sensor has Hall Effect and captures the magnetic presence of a small magnet attached to the front support roller to the treadmill belt, marking each turn full. With this measurement, the velocity has been obtained by signal conditioning to the shifting time, since the pulses are generated every complete turn of the treadmill front roller.

With the linear displacement for each turn, given by ΔS=2πr equation, we have observed by the dimensions of non-motorized treadmill that at each sensor signal the linear distance traveled 0.24 m. Thus, we could determine the running velocity by the reason of this value by the interval time between each pulse.

For power determination (Watt), the data interpretation generated from the load cell system to non-motorized treadmill has enabled the capture of the horizontal force generated by the subject. From this, in every evaluation we could obtain the mechanical power value held by the individual, through the velocity force.

The kinetic power, strength and velocity generated by each subject during the AO3 test have been recorded and analyzed in time. The common feature to this response (at least regarding power parameter) is elevated values at the beginning and fall at the last minute, showing stabilization at this stage. For determining purposes, we have considered the average values of critical power (EP) in the final thirty seconds of evaluation. Analyzing the area obtained in kinetics by trapezoidal method (power), the anaerobic protocol component has been determined, by subtracting amounts related to the supply by aerobic processes (product of time by EP). Thus, it was possible to find the anaerobic work capacity (W') values from power output graphs.

To establish the intensity of each CP test predictive load, we have calculated all graphs of horizontal power obtained in each of the four test sessions referred to as predictive load 1 (Predictive 3 elastics), predictive load 2 (Predictive 4 elastics), predictive load 3 (Predictive 5 elastics) and predictive load 4 (Predictive 6 elastics). The mechanical power values obtained have been subsequently confronted with the correspondent limit times to application of the three mathematical models: power hyperbolic vs. time limit (Hip), linear power vs. 1/time (Ԏ vs t) and linear work vs. time limit (P vs. 1/t). Through graphical analysis it was possible to obtain the aerobic (CP) and anaerobic (AWC) parameters of capacity originating from the Hip model (CP Hip and AWC Hip), from the model Ԏ vs t (CP Ԏ vs T and AWC Ԏ vs t) and model CP P vs 1/t (CP P vs 1/t and AWC P vs.1/t).

The blood lactate concentrations in test and retest of AO3 and CP have been determined by enzymatic method. The blood (25μL) collected during the assessments has been immediately transferred to 1.5 ml Eppendorf tubes containing 400 μL solution of 4% trichloroacetic acid (TCA) for deproteinization and storage at a temperature from 2 to 8°C. After that the tubes have been shaken and centrifuged for subsequent removal of 100μl of the supernatant in each sample, which have been transferred to test tubes, being added 500μl of reagent (glycine stock/EDTA), hydrazine hydrate 33%, NAD (beta-Nicotinamide Dinucleotide, SIGMA) and LDH (L-Lactic Dehydrogenase bovine heart - 1000 units/mL, SIGMA) at pH 8.85. The samples have been then shaken and incubated for 20 minutes in water bath maintained at 37ºC. Lactate concentration has been determined at 340 nm against calibration curve with standards of 5, 10, 15 and 30mM method [[3](#_ENREF_3)].

**S1 References**

1. Gama MCT, Sousa FAB, dos Reis IGM, Gobatto CA. Reliability of the Three-minute All-out Test for Non-motorized Treadmill Tethered Running. Int J Sports Med. (EFirst). Epub 13.07.2016. doi: 10.1055/s-0035-1565238.

2. Pereira VH, Gama MC, Sousa FA, Lewis TG, Gobatto CA, Manchado-Gobatto FB. Complex network models reveal correlations among network metrics, exercise intensity and role of body changes in the fatigue process. Scientific reports. 2015;5:10489. doi: 10.1038/srep10489. PubMed PMID: 25994386; PubMed Central PMCID: PMC4440209.

3. Engel PC, Jones JB. Causes and elimination of erratic blanks in enzymatic metabolite assays involving the use of NAD+ in alkaline hydrazine buffers: improved conditions for the assay of L-glutamate, L-lactate, and other metabolites. Analytical biochemistry. 1978;88(2):475-84. Epub 1978/08/01. PubMed PMID: 29519.
